# Effect of energy drink consumption on baroreceptor sensitivity in young normal weight and overweight/obese males

**DOI:** 10.12669/pjms.36.7.2419

**Published:** 2020

**Authors:** Farrukh Majeed, Talay Yar, Ahmed A Alsunni, Ali F AlHawaj, Ahmed A AlRahim

**Affiliations:** 1 Dr. Farrukh Majeed, FCPS. Department of Physiology, College of Medicine, Imam Abdulrahman Bin Faisal University, Dammam, Kingdom of Saudi Arabia; 2 Dr. Talay Yar, PhD. Department of Physiology, College of Medicine, Imam Abdulrahman Bin Faisal University, Dammam, Kingdom of Saudi Arabia; 3 Dr. Ahmed A Alsunni, PhD. Department of Physiology, College of Medicine, Imam Abdulrahman Bin Faisal University, Dammam, Kingdom of Saudi Arabia; 4 Dr. Ali F Alhawaj, MBBS. Department of Physiology, College of Medicine, Imam Abdulrahman Bin Faisal University, Dammam, Kingdom of Saudi Arabia; 5 Dr. Ahmed A AlRahim, MBBS. Department of Physiology, College of Medicine, Imam Abdulrahman Bin Faisal University, Dammam, Kingdom of Saudi Arabia

**Keywords:** Baroreceptor sensitivity, Energy drink, Obesity, Caffeine

## Abstract

**Objectives:**

There is lack of evidence exploring sympathetic effect by baroreceptor sensitivity in obese consuming energy drink. The purpose of this study was to investigate the acute effect of energy drink on individuals baroreceptor sensitivity in young healthy normal weight and overweight/obese males.

**Methods:**

This cross-sectional study was performed in the Department of Physiology, Imam Abdulrahman Bin Faisal University, Kingdom of Saudi Arabia. After getting ethical approval, 25 male participants were recruited by convenient sampling and informed consent was obtained. Participants were grouped into normal weight and overweight/obese on basis of body mass index. Finger arterial blood pressure was recorded with Finometer® at baseline, 30min and 60 minutes in the post-energy drink period and baroreceptor sensitivity was calculated. As data was not normally distributed it was log transformed.

**Results:**

The baseline baroreceptor sensitivity was lower (P<0.05) in overweight/obese compared to normal weight participants. Baroreceptor sensitivity reduced significantly (P<0.05) at 60 minutes after energy drink consumption in the whole cohort of both normal weight and overweight/obese. Baroreceptor sensitivity remained lower in overweight/obese compared to normal weight at 60min but the difference was not significant.

**Conclusion:**

Consumption of energy drink acutely reduced baroreceptor sensitivity in both normal weight and obese young healthy males with an earlier onset of effect in overweight/obese indicating enhanced sympathetic activity. Energy drinks consumption could place the obese in a more vulnerable state to hypertension and arrhythmia.

## INTRODUCTION

Energy drinks (ED) are highly caffeinated drinks that are more popular in young adults for improving alertness, enhancing memory but at the cost of reported adverse consequences.^1^ Recent studies on ED raised concerns about their cardiovascular complications especially in frequent consumers such as individuals from young age group or those with underlying cardiovascular disease.^2^ Obese individuals are more prone to variety of adverse cardiovascular events such as hypertension, myocardial infarction, arrhythmia and sudden death.^3^ Compared to normal weight individuals the obese individuals exhibit autonomic imbalance manifested as enhanced resting sympathetic activity, lower parasympathetic activity and a lower spontaneous baroreceptor sensitivity (BRS).^4^ This autonomic imbalance could underlie the higher morbidity and mortality in obese individuals.^5^

Arterial blood pressure is regulated on a beat to beat basis with the help of baroreceptors located in carotid bodies and aortic arch. With elevation of blood pressure these baroreceptors activate the parasympathetic system and concurrently inhibit the sympathetic system leading to reduction in vascular resistance and decrease in heart rate.^6^ This helps to bring the blood pressure back to normal range. This ability of baroreceptors to respond reflexly to changes in blood pressure and modulate the interbeat interval (inverse of heart rate) is termed as baroreceptor sensitivity (BRS). A higher BRS (expressed as msec/mmHg) indicates a better control of blood pressure and is considered a better prognostic feature in cardiovascular diseases.^7^ Reduced BRS, as might result in sympathetic activation because of a variety of reasons would lead to a greater vulnerability to cardiovascular morbidities. In addition to the blood pressure (BP) changes in response to various types of physical or mental stresses, there are also spontaneous fluctuations in BP and BRS under resting conditions. This spontaneous fluctuation in the activity of baroreceptors is termed as spontaneous BRS and is a valuable non-invasive tool to assess how efficiently the baroreflex buffers the beat-to-beat variations in blood pressure.^8^ BRS has been shown to be influenced by age, gender and obesity level.^9^,^10^

Effects of ED consumption could be accentuated in obese individuals who are prone to autonomic dysfunction. Though alteration in autonomic balance has been reported in overweight/obese individuals as well as in consumers of energy drinks, there are hardly any investigations studying the effects of energy drink consumption on BRS in overweight/obese.^11^,^12^

We hypothesized that the individuals spontaneous BRS will be less in young overweight/obese compared to normal weight individuals at baseline, and the consumption of ED will have a greater depressant effect on BRS in overweight/obese compared to normal weight.

The objective of the present study was to noninvasively measure the spontaneous BRS in young healthy normal weight and overweight/obese males and compare the acute effect of ED consumption on BRS in normal weight and overweight/obese groups.

## METHODS

Permission and ethical approval for this study was obtained from deanship of scientific research, (Grant No.: 2012098, IRB-213-24-1, November 2013) Imam Abdulrahman Bin Faisal University in Kingdom of Saudi Arabia. This study was carried out in the period between December 2013 and December 2014.

Twenty-five male students from the university were recruited by convenient sampling and grouped into normal weight (n=13) and overweight/obese (n=12) based on body mass index (BMI). The inclusion criteria for study participants were: 18-22 years old male university students, non-smokers and who were not regular users of ED. Exclusion criteria were the presence of chronic medical condition, regular consumption of herbal medications, known sensitivity to taurine or caffeine and trained athletes.

The participants were briefed about the project in a familiarization session and written consent was taken. Participants were instructed to avoid caffeine for at least three days and report with 12 hours fasting at their allotted time.

### Sample size calculation

For sample size calculation, OpenEpi, version 3, open-source calculator was used. Based upon a pilot study on five volunteers (the results of whom were not included in the final data) we assumed that mean BRS in normal weight group will be 11.5 (±2.56) ms/mmHg and it will be 8.48 (±2.6) ms/mmHg in the overweight/obese group. At a confidence interval of 95 %, power of 80% and at an alpha level (P–value) of 0.05 we calculated the sample size to be 11 in each group.

### Anthropometric measurement

Weight in kilogram was assessed in light clothing and without shoes to the nearest 0.5 kg using digital scale. Height was measured to the nearest 0.5 centimeter in upright standing posture using a stadiometer. BMI was calculated and participant were categorized as normal weight (BMI =18.5-24.9 kg/m^2^) and overweight/obese (BMI ≥25 kg/m^2^).^13^

### Baroreceptor sensitivity measurement

Finometer® Pro (Finapress Medical Systems, Amsterdam, Netherlands) was used to record beat-to-beat finger arterial blood pressure. Finometer uses the volume clamp technique to measure finger arterial pressure by photo-plethysmopgraphy.^14^

The finger arterial blood pressure data was analyzed with the help of Beatscope® and PRVBRS software (Finapres Medical Systems, The Netherlands). The spontaneous BRS during resting condition was estimated by cross correlation time-domain method that analyzed the relationship between beat-to-beat blood pressure and the inter beat interval. The program detects the sequence of at least three successive beats where there is a spontaneous increase in blood pressure of at least one mmHg every beat that leads to an increase of at least five milliseconds in the interbeat interval (hypertension/bradycardia), or a reduction in blood pressure leading to a reduction in interbeat interval (hypotension/tachycardia). The mean regression slopes of all the sequences are averaged to calculate the BRS.^15^

After baseline recording for 10-15 minutes, participants took the energy drink (5ml/kg body weight; a can contains taurine 100mg, glucronolactone 600mg, caffeine 80mg, vitamin B5 6mg, sucrose 21.5g and glucose 5.25gm). Further recording was done at 30 and 60 minutes after ED consumption. The whole session took approximately one hour thirty minutes for each individual. Participants with inappropriate/incomplete data were not included in the final analysis. It was not possible to obtain BRS data in four participants and for one participant a technical error led to inability to obtain BRS data. Therefore, the BRS data presented is only from 20 participants including 11 normal weight and nine overweight /obese (two overweight and seven obese). It has been documented that BRS may not be measurable in some participants who are otherwise normal but do not show significant spontaneous concordant fluctuation in BP and heart rate.^16^

### Statistical Analysis

Statistical analysis was performed using the SPSS statistical package version 20.0 (IBM Corp., Armonk, N.Y., USA). The normality of data was tested by the Shapiro-Wilk test and BRS data was found to be skewed. Therefore, the BRS data was natural log transformed (lnBRS) for further analysis with parametric tests.^17^ Data are presented as means ±SD (standard deviation). The comparison of data between normal weight and overweight/obese groups was performed by independent sample t-test, while one way Analysis of variance (ANOVA) with post hoc Bonferroni were used for comparison between individual groups at baseline (0 minute), 30 and 60 minutes. The association between the lnBRS and BMI was calculated by Pearson’s correlation coefficient. For all the tests, a P-value of <0.05 was considered statistically significant.

## RESULTS

The baseline demographic characteristics of two groups are presented in Table-I. As expected, the BMI, was significantly higher (P< 0.001) in overweight/obese group. The mean±SD of spontaneous BRS in normal weight was 11.21±2.25 msec/mmHg whereas in the overweight/obese it was 9.19±2.52 msec/mmHg) (Table-I).

**Table-I:** Baseline characteristics of study participants.

	Normal weight (n=12) Mean± Standard Deviation	Overweight /Obese (n=13) Mean± Standard Deviation	P value
Age (years)	20.6±0.6	20.5±0.7	0.523
Body mass index (kg/m^2^)	22.3±2.1	34.2±5.4	0.000*
Systolic blood pressure (mm Hg)	129.6±9.1	131.8±11.8	0.646
Diastolic blood pressure (mm Hg)	79.0 ±13.6	82.8±8.2	0.467
Heart rate (beats/min)	76.9±9.3	78.6±9.9	0.692
Spontaneous Baroreceptor sensitivity (msec/mmHg) #	11.21 ±2.2	9.19 ±2.5	0.075

# n=20 (normal weight=11, Overweight/obese=9),

* P <0.001

**Fig.1:**
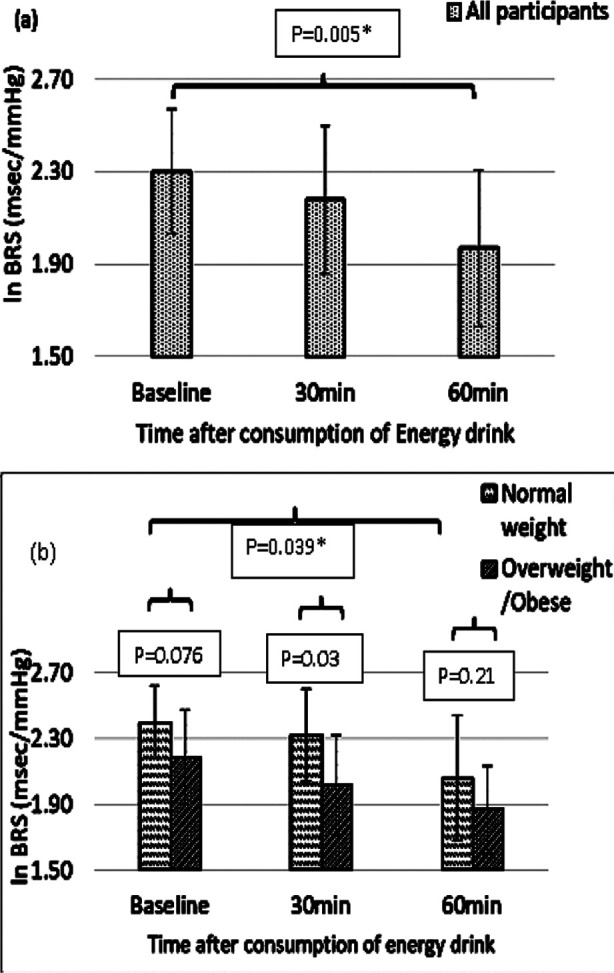
**a)** Resting lnBRS in the whole cohort (n=20) and the effect of energy drink at baseline (0 minute), 30 and 60 minutes after energy drink consumption. **b)** Comparison of lnBRS between normal weight (n=11) and overweight/obese (n=9) groups and within the group, at baseline (0 minute) and at 30 and 60 minutes after energy drink consumption. *lnBRS= natural log transformed baroreceptor sensitivity.

One way ANOVA revealed that ln BRS in the whole cohort of (20) participants was significantly reduced (F=5.69, P=0.006). Posthoc with bonferroni (Fig.1a) revealed that the significant difference was at 60 minutes of energy drink consumption compared to baseline (P=0.005). Furthermore, normal weight group (n=11) showed a significant reduction (F=3.81, P=0.033) in lnBRS after consumption of energy drink. Posthoc with bonferroni revealed that the significant difference (P=0.039) was at 60 minutes after energy drink consumption compared to baseline (Fig.1b). The lnBRS in overweight/obese group (n=9) did not show a statistically significant change with respect to time (F=2.75, P=0.084). Comparison between normal weight and overweight/ obese group showed that lnBRS was significantly less in overweight/obese at 30 minutes (P=0.032) while no statistically significant difference was observed either at baseline (P=0.076) or at 60 minutes (P=0.21) after energy drink consumption (Fig.1b).

A significant negative correlation (r = -0.488, P= 0.029) of BMI with lnBRS at 30 minutes was observed, while other findings were statistically insignificant at base line (r = -0.385, P=0.093) and 60 minutes (r = -0.258, P= 0.275).

## DISCUSSION

To the best of our knowledge this is the first study in Saudi Arabia to measure the BRS in young individuals. We have demonstrated that (i) the spontaneous BRS was significantly reduced after ED consumption in the whole cohort of these normal weight and overweight/obese young participants and (ii) spontaneous BRS was significantly less in young overweight/obese males compared to normal weight at 30 minutes after consumption of energy drink.

Changes in the baroreceptor-heart rate reflex contribute to the imbalance between sympathetic and parasympathetic system, and measurement of BRS is a source of important information in the clinical management of patients suffering from cardiac disease and hypertension.^7^,^18^ A reduced BRS is strongly associated with hypertension.^19^ The BRS reduction in overweight/obese at 30 minutes was significant, indicating an earlier effect of ED in the overweight/obese group compared to normal weight. At 60 minutes the reduction in BRS was slightly greater in overweight/obese compared to normal weight but the difference was not significant. One possibility of non-significance difference at 60 minutes could be the small sample size and need to be studied further. Another possibility is that all our participants were otherwise healthy and did not have high blood pressure and most likely had well preserved baroreceptor function. A value of BRS greater than 6ms/mmHg is taken as an indication of well-preserved autonomic function. If BRS is less than 3ms/mmHg it is considered as depressed autonomic function whereas a BRS of greater than 3ms/mmHg is taken as preserved autonomic balance.^16^ We have thus extended the observation of other investigators to include Arab ethnicity. Ethnic differences are known to exist in autonomic balance that is reflected in heart rate variability and BRS.^20^,^21^

Animal and human studies suggest that BRS is attenuated whenever sympathetic activity is enhanced.^5^ ED dependent sympathetic augmentation has been demonstrated by many investigators and possible suggested mechanism of this effect include promoting atherosclerosis, enhancing platelet aggregation, triggering endothelial dysfunction and inducing a positive inotropic effect on cardiac functions.^22^,^23^

Our study confirms and extends the earlier finding by Indumathy et al, Skrapari et al, Lazarova et al, who described negative correlation of BMI with BRS in obese and pre-obese individuals.^4^,^24^,^25^ A number of possible mechanisms for reduction of BRS in obese have been suggested including thickening of intima media of carotid vessels in obese.^26^ A high plasma leptin in obese rats was observed to depress the response of nucleus tractus solitarius to aortic baroreceptor stimulation and hampering the baroreceptor reflex.^27^ Both diet-induced and exercise induced weight loss in obese individuals has been associated with improvement in autonomic balance manifested as sympatho-inhibition and/or enhanced vagal modulation of heart and have been linked with improved outcomes after cardiac events including sudden death.^9^,^28^ Although some studies have examined the autonomic disturbances and altered BRS caused by other diseases, there is scarcity of data investigating the effects of ED on BRS.^29^,^30^

The synergistic acute effects of ED and obesity on BRS have previously not been assessed in humans. The present study is the first to demonstrate a significant attenuation of BRS by ED. Both caffeine and sugar content could be the reason for enhancement of activation of the SNS that could depress BRS in young male individuals. Thus, the consumption of energy drinks could put the young individuals especially the obese ones at a greater risk of developing hypertension and arrhythmias. The results of present study could help in further raising public awareness about harmful effects of ED and stimulating lifestyle modifications such as reducing the consumption of ED, and attaining and maintaining healthy weight. The information might also be useful in field of public health and formulation of policies related to these beverages.

### Limitations of the study

One of the limitations of the present study is that we observed the participants for one hour only post-consumption and thus we cannot comment on for how long the adverse effects of ED on autonomic balance would persist. Our results are also limited to demonstration of acute changes in autonomic balance induced by ED. Further studies scrutinizing chronic effects of ED consumption are recommended. Finally, our sample size is small to derive a strong conclusion. Thus, the inclusion of more participants is recommended to improve the power of future studies.

## CONCLUSIONS

Energy drinks induced a significant reduction in BRS in all the young participants but because of a probable higher baseline sympathetic activity the effect was exhibited earlier in the overweight/obese group compared to normal weight. ED consumption might put the young individuals especially the overweight/obese at a greater risk of developing hypertension and other cardiac disturbances. Concerted efforts are required towards cautioning the public at large (normal weight & overweight/obese) about intake of ED.

### Authors’ Contribution:

**FM:** conceived, designed and did statistical analysis, editing of manuscript responsible for accuracy and integrity of work.

**TY:** conceived, designed and did statistical analysis and editing of manuscript.

**AAA:** did data collection and data acquisition and manuscript editing.

**AFA:** did data collection and data acquisition and manuscript editing.

**AAA:** did review and final approval of manuscript.
